# Supplementary material for: MR-SPLIT: a novel method to address selection and weak instrument bias in one-sample Mendelian randomization studies
Source: PLoS Genet. Author manuscript; Available in PMC 2024 Oct 28. (PMC11410202; doi:10.1371/journal.pgen.1011391)
Supplement: Supplementary material [file EMS198662-supplement-Supplementary_material.pdf]

Supplementary Materials for
"MR-SPLIT: a novel method to address selection and weak instrument bias in one-sample Mendelian randomization studies"

Ruxin Shi[1], Ling Wang[2], Stephen Burgess[3] and Yuehua Cui[1*]

1 Department of Statistics and Probability, Michigan State University, East Lansing, Michigan, USA
2 Department of Medicine, Michigan State University, East Lansing, Michigan, USA
3 Biostatistics Unit, University of Cambridge, Cambridge, UK

*Corresponding author: cuiy@msu.edu

This supplementary file contains additional simulation and real data analysis results. The proof of Theorem 1 is given at the end of this file.

1 Results of selecting major IVs under different partial F values

Table S1 shows the results of distinguishing major and weak IVs with different partial F thresholds. We also showed the results with the criterion of $F > 100$. As demonstrated, this is exceedingly conservative and is generally best avoided. Furthermore, we recognize that a heritability ($h^2 = 0.5$) is considerably high for many exposure traits in practical scenarios, representing situations that are relatively uncommon in reality.

Table S1: Mean numbers of being identified as major IV using different criteria in 1000 simulations. For the noise category, it was aggregated over the 295 null IVs.

h^2	N	Criteria	SNP_1	SNP_2	SNP_3	SNP_4	SNP_5	Noises ($\times$295)
0.15	500	F>10	0.55	0.5	0.15	0	0.1	1.25
		F>30	0.05	0.05	0	0	0	0
		F>50	0	0	0	0	0	0
		F>100	0	0	0	0	0	0
	1000	F>10	0.95	0.95	0.35	0.1	0	0.65
		F>30	0.35	0.35	0	0	0	0
		F>50	0.15	0	0	0	0	0
		F>100	0	0	0	0	0	0
	2000	F>10	1	1	0.75	0.35	0.55	0.6
		F>30	1	0.95	0.1	0	0	0
		F>50	0.75	0.75	0	0	0	0
		F>100	0	0	0	0	0	0
0.3	500	F>10	0.95	0.8	0.25	0.25	0.1	1.15
		F>30	0.5	0.55	0	0	0	0
		F>50	0.25	0.25	0	0	0	0
		F>100	0	0	0	0	0	0
	1000	F>10	1	1	0.75	0.45	0.3	0.65
		F>30	1	1	0.2	0	0	0
		F>50	0.8	0.7	0.05	0	0	0
		F>100	0	0.05	0	0	0	0
	2000	F>10	1	1	1	0.75	0.9	0.6
		F>30	1	1	0.6	0.15	0.1	0
		F>50	1	1	0.1	0	0.05	0
		F>100	1	1	0	0	0	0
0.5	500	F>10	1	1	0.8	0.35	0.4	1.8
		F>30	1	1	0.35	0	0.05	0
		F>50	0.9	0.9	0	0	0	0
		F>100	0.2	0.3	0	0	0	0
	1000	F>10	1	1	1	1	0.95	0.6
		F>30	1	1	0.8	0.5	0.15	0
		F>50	1	1	0.4	0.05	0	0
		F>100	1	1	0.05	0	0	0
	2000	F>10	1	1	1	1	1	0.4
		F>30	1	1	1	0.9	0.9	0
		F>50	1	1	1	0.4	0.3	0
		F>100	1	1	0.35	0	0	0

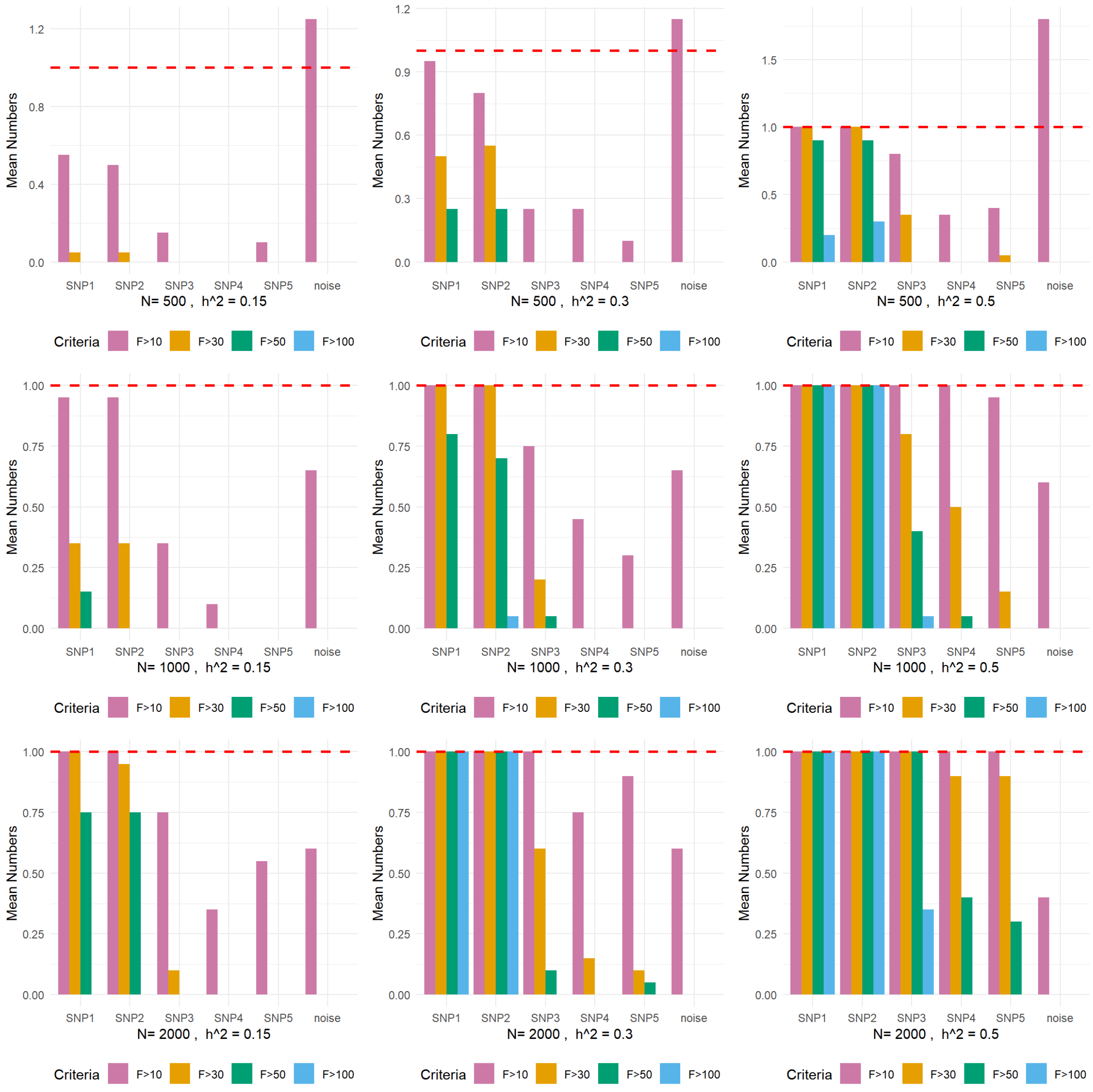

Figure S1: Mean numbers of being identified as major IV using different thresholds in 1,000 simulations.

2 Boxplots of causal effect estimates for MR-SPLIT, 2SLS, and LIML out of 1000 simulation runs under different scenarios.

LIML and 2SLS both use half of the dataset to select IVs and the other half to get the estimation. In contrast, LIML_w and 2SLS_w use the whole dataset for IV selection and causal effect estimation. When using half data to select IVs and another half for causal estimation, both MR-SPLIT and LIML produce unbiased estimates, though the variance for LIML is larger than MR-SPLIT. However, when using the whole data for both IV selection and causal effect estimation, LIML_w and 2SLS_w generate biased causal effect estimation. In either case, 2SLS yields biased effect estimates. This simulation demonstrates the issue of IV selection bias if it is not properly addressed.

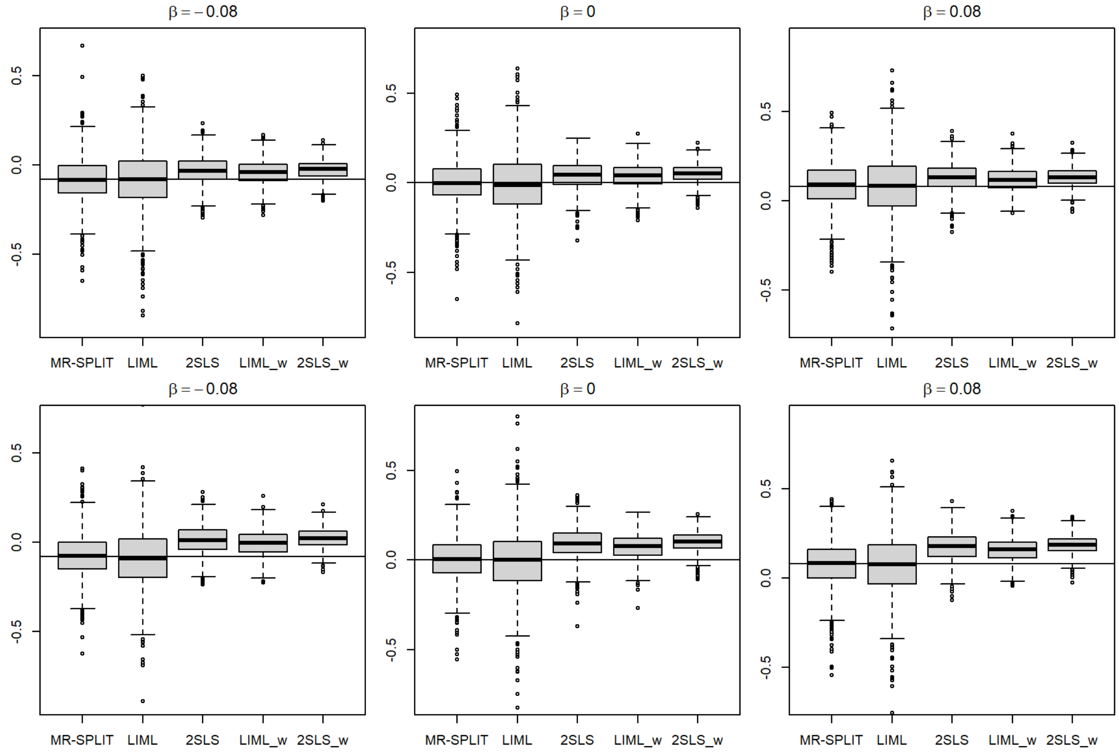

Figure S2: Boxplots of causal effect estimates ($\hat{\beta}$) under $h^2 = 0.15$ and confounding correlation $\rho = 0.1$ (top) and $\rho = 0.2$ (bottom).

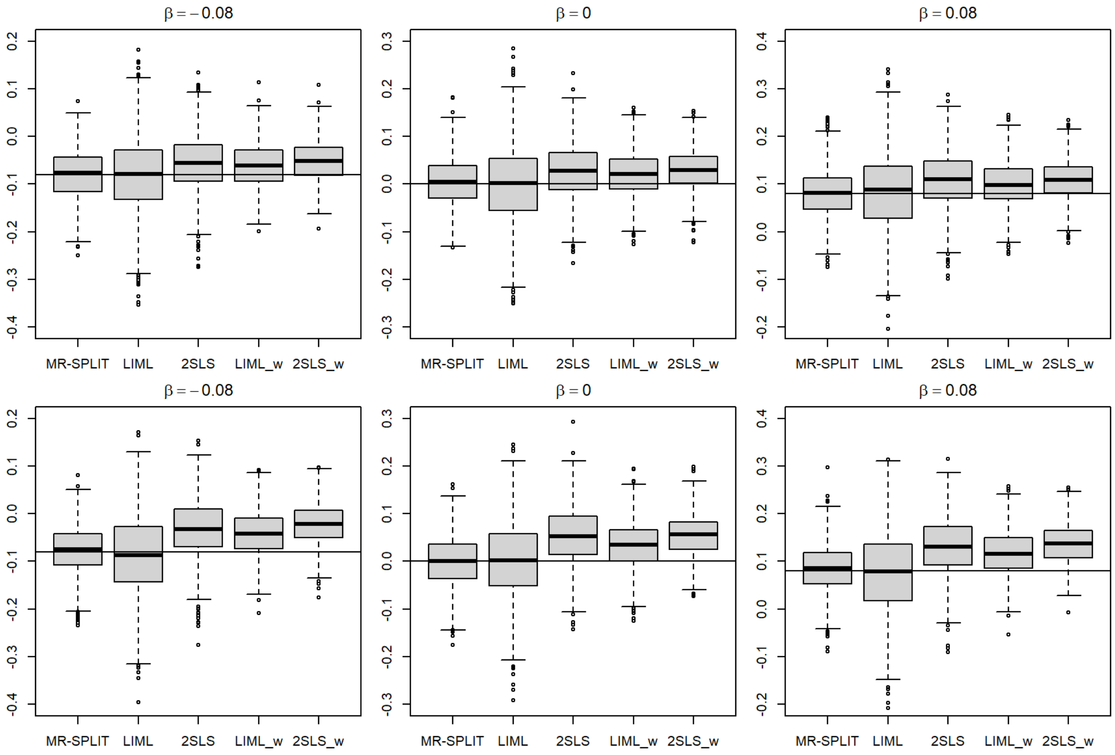

Figure S3: Boxplots of causal effect estimates ($\hat{\beta}$) under $h^2 = 0.3$ and confounding correlation $\rho = 0.1$ (top) and $\rho = 0.2$ (bottom).

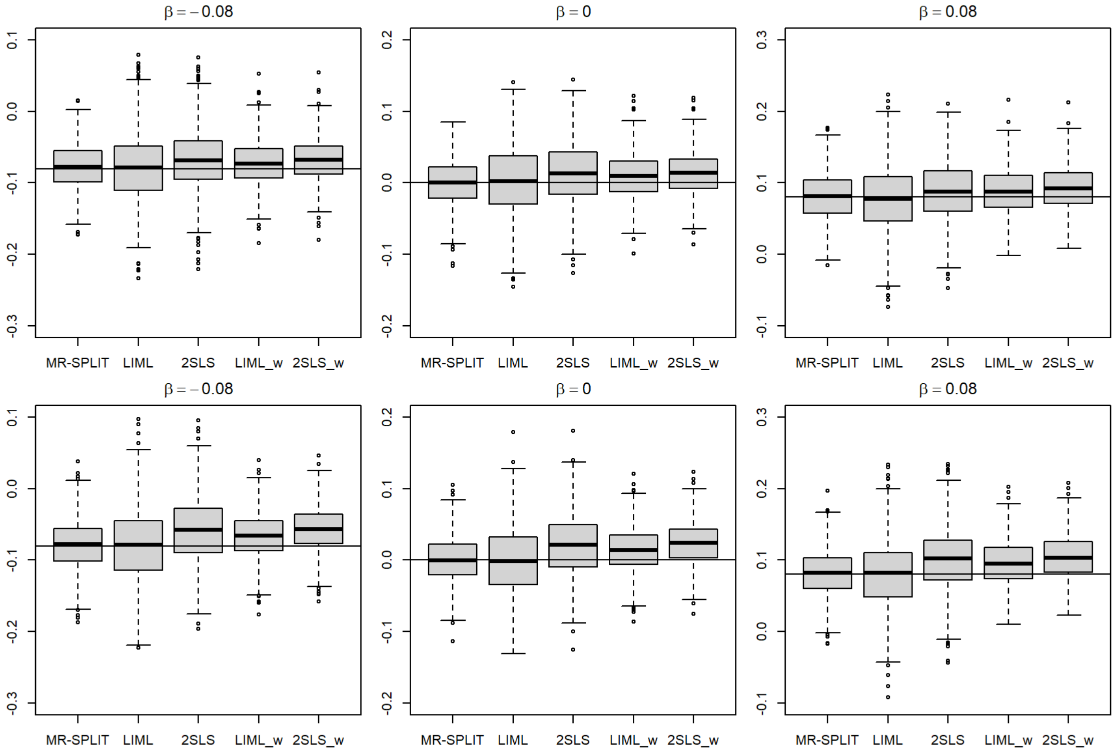

Figure S4: Boxplots of causal effect estimates ($\hat{\beta}$) under $h^2 = 0.5$ and confounding correlation $\rho = 0.1$ (top) and $\rho = 0.2$ (bottom).

3 Boxplots of causal effect estimates for MR-SPLIT and CFMR out of 1000 simulation runs under different scenarios.

In nearly all scenarios, both CFMR and MR-SPLIT obtained approximately unbiased estimates. However, it is evident that MR-SPLIT consistently exhibits a smaller variance compared to CFMR. This can also be seen in the comparison of RMSE (see Figure S11), where the RMSE of MR-SPLIT is always noticeably smaller than that of CFMR.

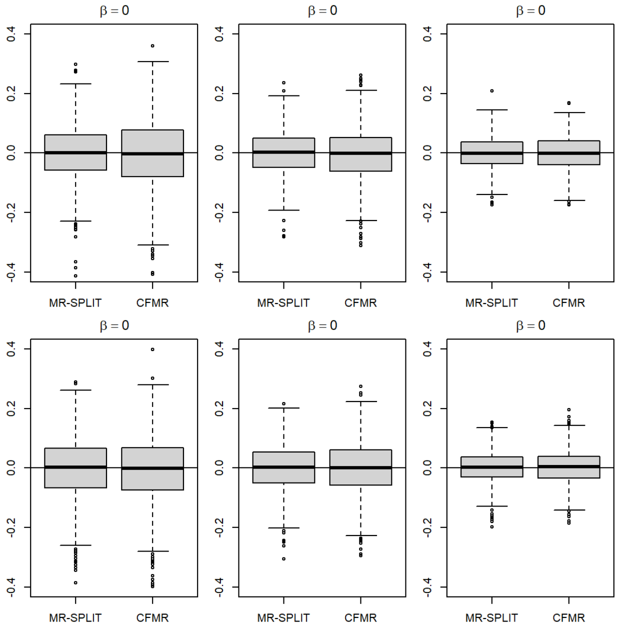

Figure S5: Boxplots of causal effect estimates ($\hat{\beta}$) when $h^2 = 0.15$ (left), 0.2 (middle) , 0.3 (right) and sample size $N = 1000$ in scenario I (top) and scenario II (bottom).

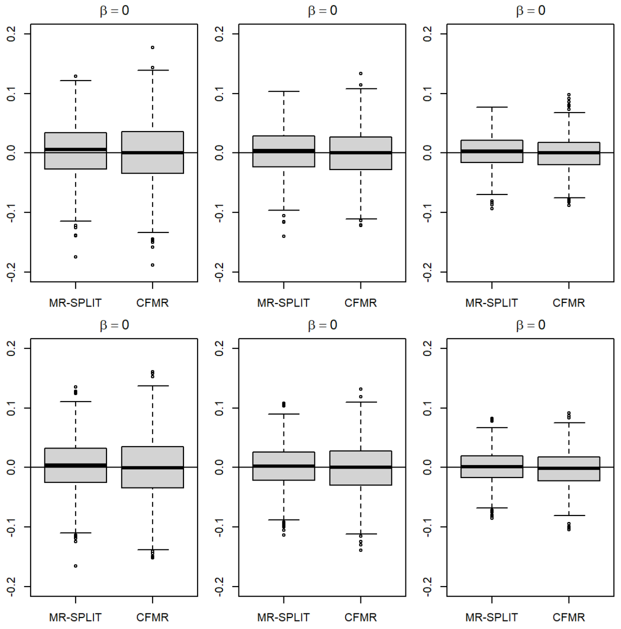

Figure S6: Boxplots of causal effect estimates ($\hat{\beta}$) when $h^2 = 0.15$ (left), 0.2 (middle) , 0.3 (right) and sample size $N = 3000$ in scenario I (top) and scenario II (bottom).

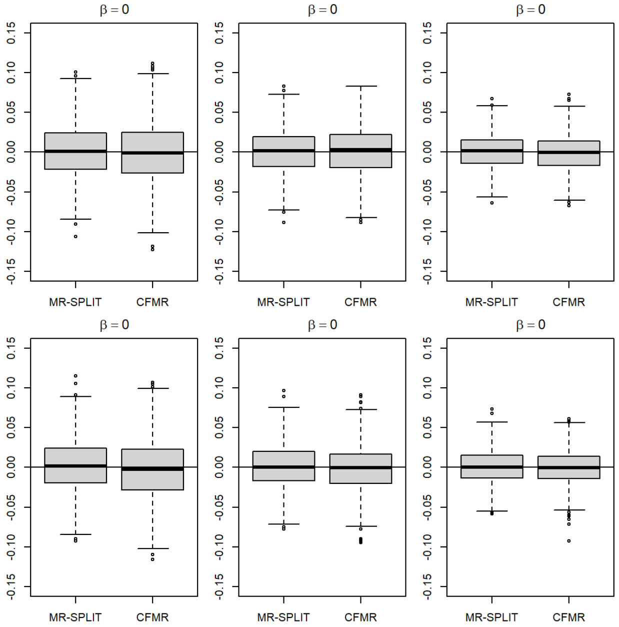

Figure S7: Boxplots of causal effect estimates ($\hat{\beta}$) when $h^2 = 0.15$ (left), 0.2 (middle) , 0.3 (right) and sample size $N = 5000$ in scenario I (top) and scenario II (bottom).

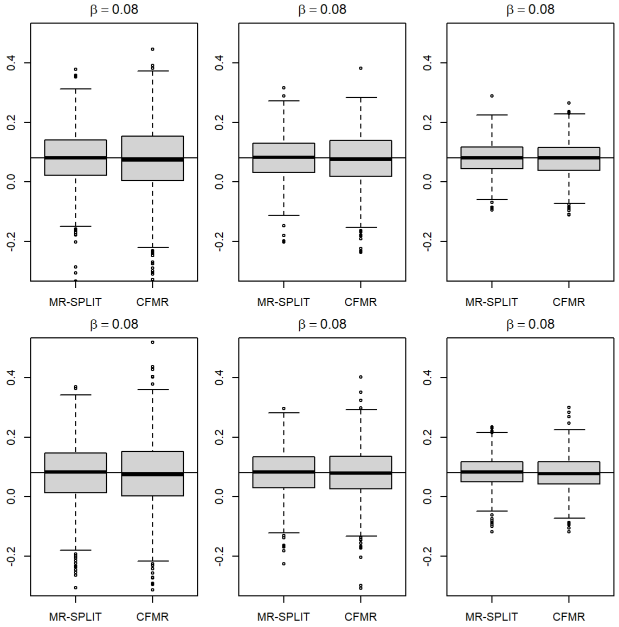

Figure S8: Boxplots of causal effect estimates ($\hat{\beta}$) when $h^2 = 0.15$ (left), 0.2 (middle) , 0.3 (right) and sample size $N = 1000$ in scenario I (top) and scenario II (bottom).

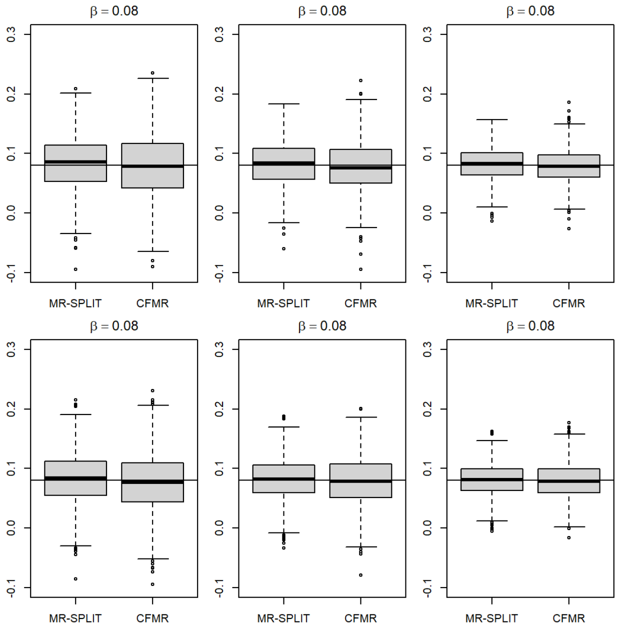

Figure S9: Boxplots of causal effect estimates ($\hat{\beta}$) when $h^2 = 0.15$ (left), 0.2 (middle) , 0.3 (right) and sample size $N = 3000$ in scenario I (top) and scenario II (bottom).

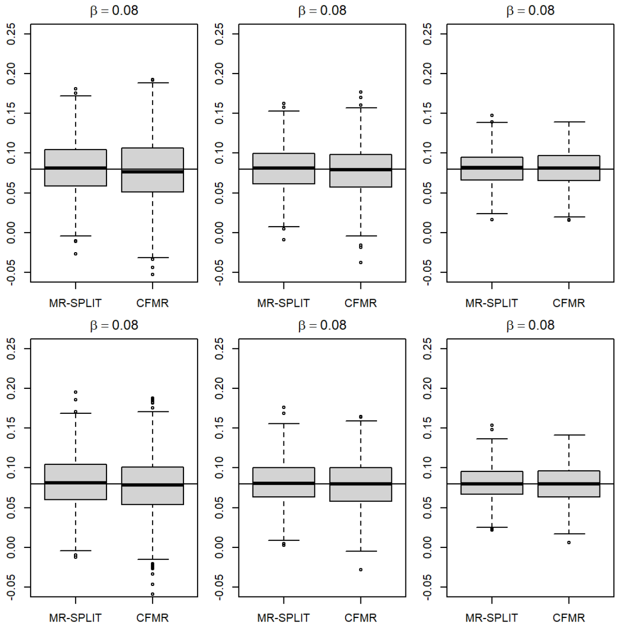

Figure S10: Boxplots of causal effect estimates ($\hat{\beta}$) when $h^2 = 0.15$ (left), 0.2 (middle) , 0.3 (right) and sample size $N = 5000$ in scenario I (top) and scenario II (bottom).

4 RMSE comparison between MR-SPLIT and CFMR out of 1000 simulation runs under different scenarios.

The RMSE of MR-SPLIT is always smaller than that of CFMR, especially under a small sample size (e.g., $N = 1000$), indicating the estimation efficiency and consistency of MR-SPLIT compared to CFMR.

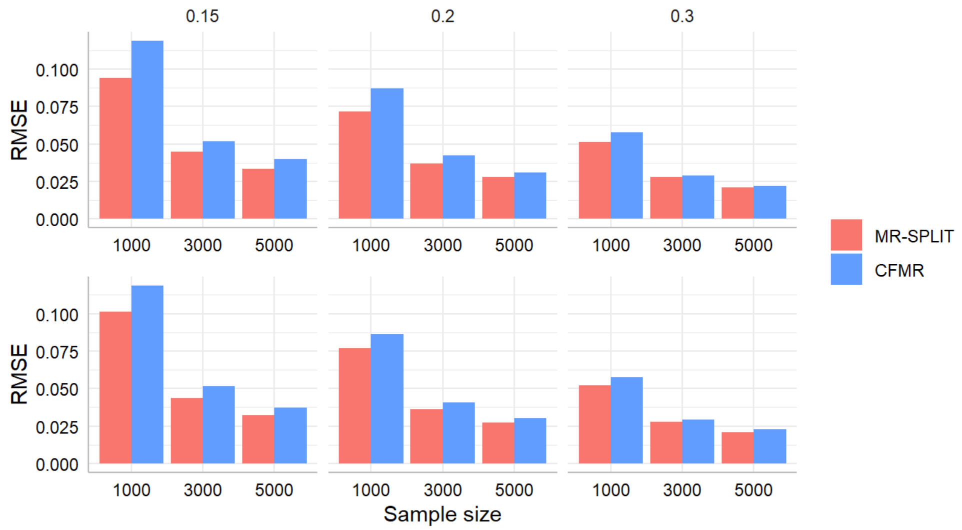

Figure S11: RNSE comparison between MR-SPLIT and CFMR in Scenario I (top) and II (bottom).

5 Additional type I error and power simulation results for the evaluation of multiple data splitting

Figure S12 shows the type I error out of 50 sample splits under $h^2 = 0.3$ and different sample sizes. When SNP heritability is significant and the sample size is relatively large (for instance, greater than 1000), the type I error stabilizes, even with a minimal number of sample splits.

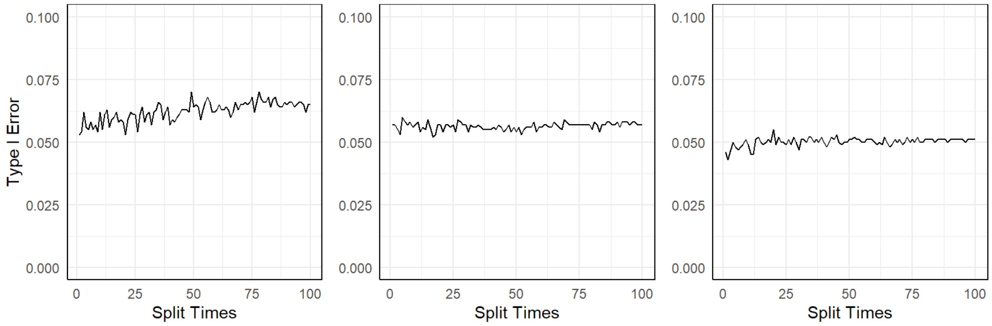

Figure S12: Type I error when $h^2 = 0.3$ and $N = 500$ (left), 1000 (middle), 2000 (right) out of 50 sample splits.

Figure S13 displays the empirical power under different sample sizes when $h^2 = 0.3$. Compared to scenarios where $h^2 = 0.15$ or 0.2, fewer splits are required to achieve optimal power. When the sample size is relatively small, for instance, $N = 500$, the power stabilizes after about 25 sample splits. As the sample size increases to 1000, a few sample splits are good enough to achieve stable power. The results suggest that in practice, one can lower the number of sample slits if the estimated SNP heritability for the exposure is strong and the sample size is large, to save computational time.

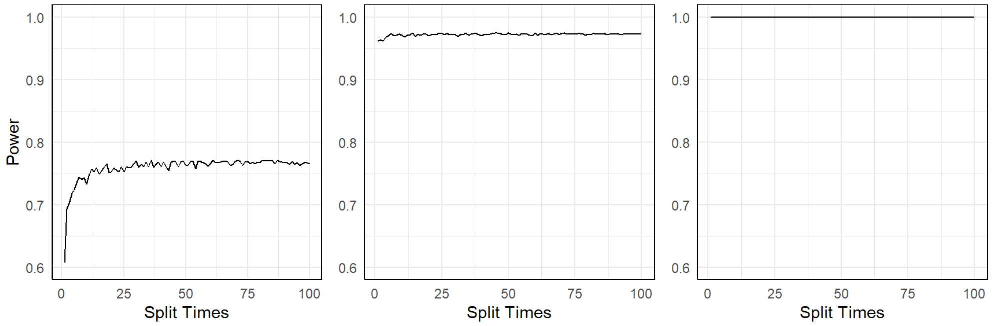

Figure S13: Power performance when $h^2 = 0.3$ and $N = 500$ (left), 1000 (middle), 2000 (right) out of 50 sample splits.

6 Comparison between the LASSO and LASSO-projection methods.

In this simulation, we used two different methods, LASSO and LASSO-projection, to do the IV selection. We considered the case with the sample size as $N = 1,000$, and randomly generated a set of 300 independent SNPs with their minor allele frequency fixed as 0.3 for all the SNPs. We randomly chose 5 SNP IVs to generate the exposure variable. The correlation between the error terms is set to 0.16. The variation in the exposure explained by the 5 SNPs is set to $h^2 = 0.2$. The real causal effects β were set to $\{-0.08, 0, 0.08\}$. Both methods produced very similar effect estimates as revealed by the boxplots in Figure S14. The LASSO-projection method yielded smaller type I error (Figure S15, slightly larger power (Figure S16 and smaller RMSE (Figure S17), compared to the regular LASSO method. We also observed that the total number of IVs selected by the regular LASSO method is much larger than the LASSO-projection method (Figure S18), and the number of major IVs selected by the LASSO-projection method is slightly higher than the LASSO method (Figure S19). We observed similar trends under other settings and hence only reported the results of this scenario.

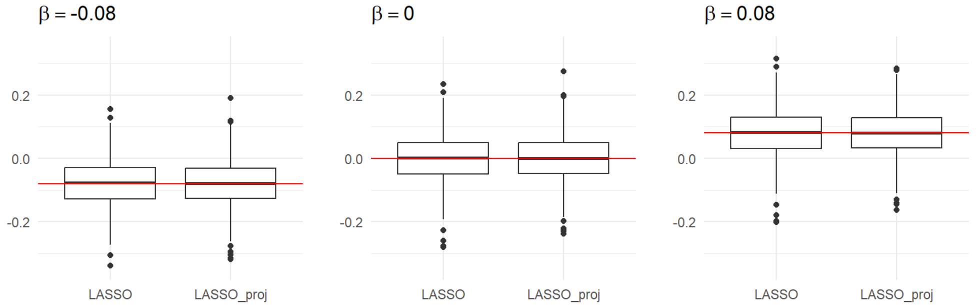

Figure S14: Boxplots of LASSO and LASSO-projection estimators.

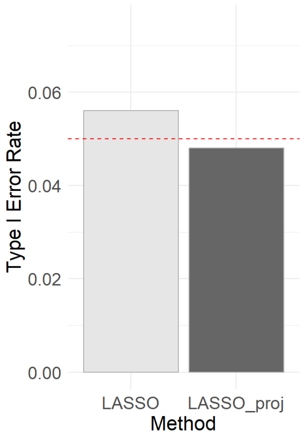

Figure S15: Type I Error.

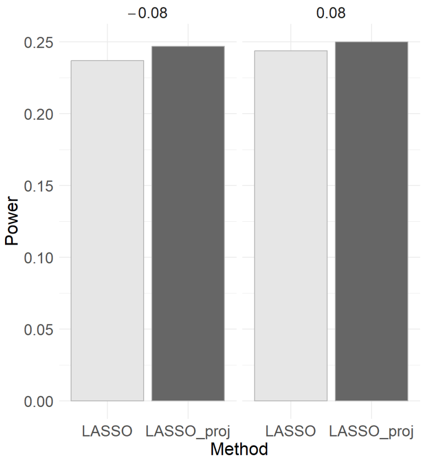

Figure S16: Power.

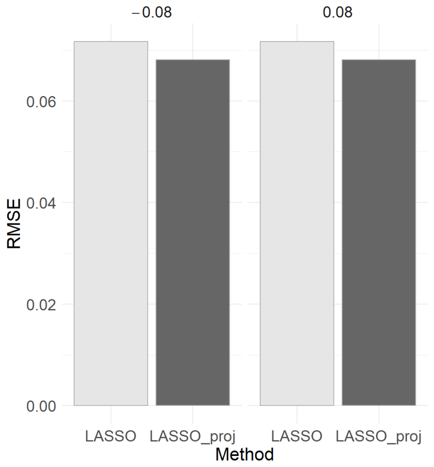

Figure S17: RMSE.

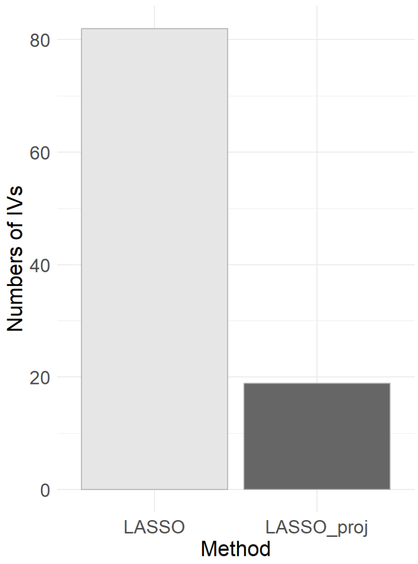

Figure S18: Total IVs selected.

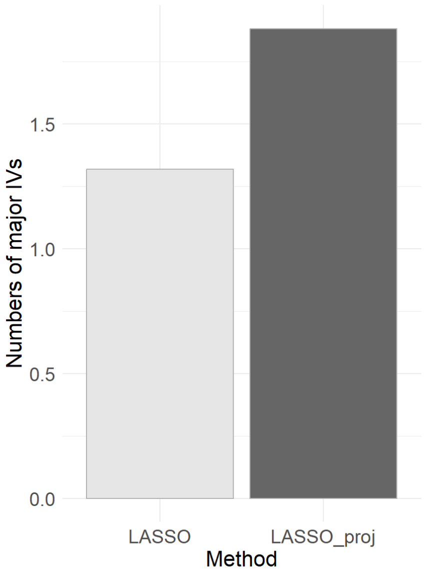

Figure S19: Major IVs selected

7 Additional results for the real data analysis

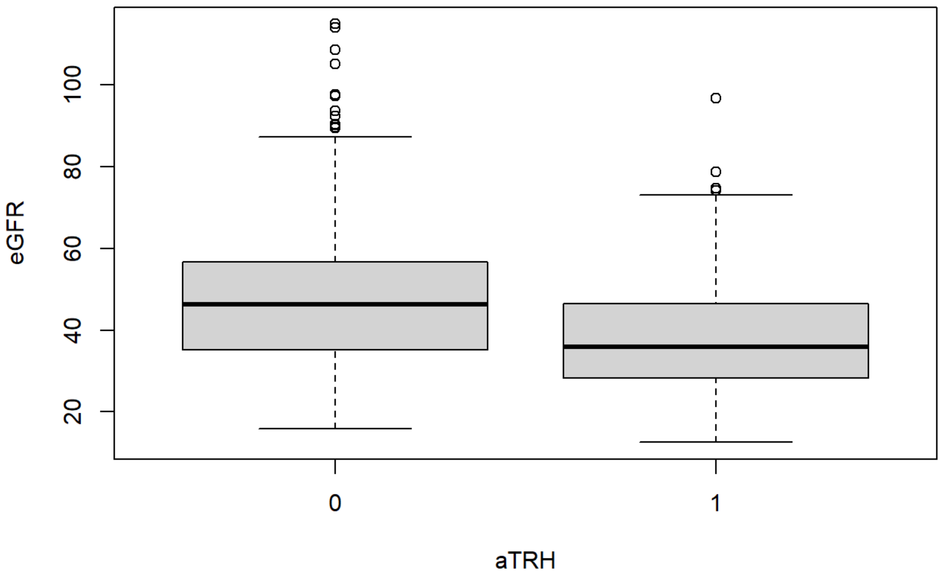

Figure S20: Boxplot of eGFR in aTRH positive and negative groups.

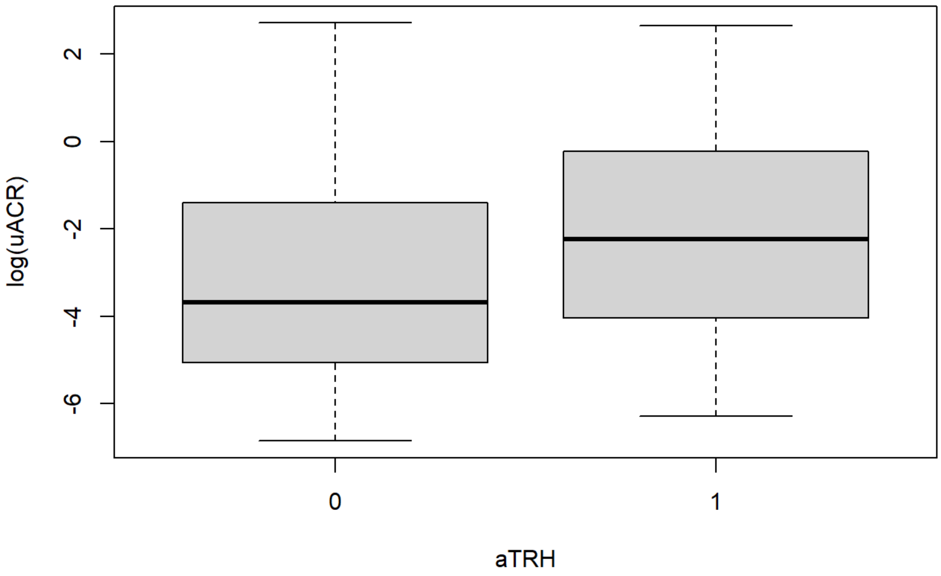

Figure S21: Boxplot of log(uACR) in aTRH positive and negative groups.

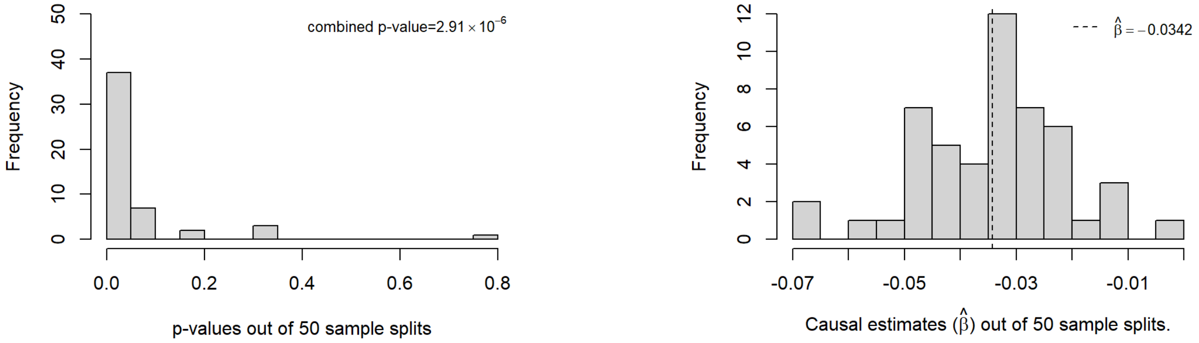

Figure S22: Histogram of p-values and causal effect estimates from 50 sample splits when eGFR is treated as the exposure (F>20 is used to distinguish the major IVs).

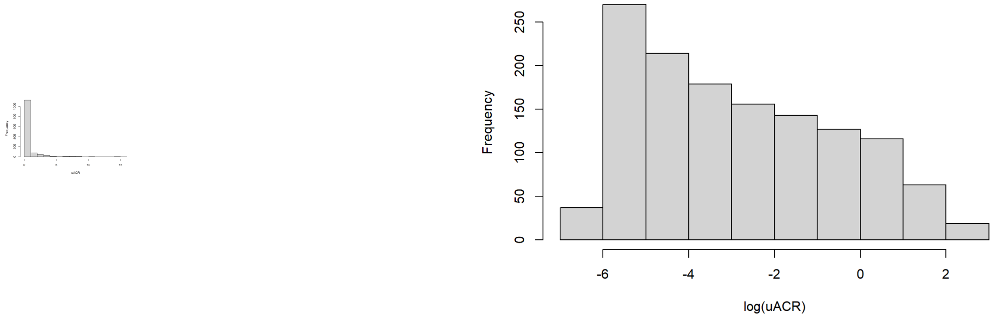

Figure S23: Histogram of uACR (left figure) and log(uACR) (right figure).

8 Proof of Theorem 1

Proof of Theorem For a given sample $\{X, Y, G\}$, the two stage IV model is defined as,

$$\begin{aligned} X &= G\alpha + \varepsilon_1 \\ Y &= X\beta + \varepsilon_2 \end{aligned} \tag{S1}$$

where $(\varepsilon_1, \varepsilon_2)' \sim N(0, \sigma^2(\begin{array}{cc} 1 & \rho \\ \rho & 1 \end{array}))$ and the correlation ρ reflects the degree of confounding effect.

Suppose we split the data into two parts, $I_1 = \{X_1, Y_1, G_1\}$, and $I_2 = \{X_2, Y_2, G_2\}$. Each subset has equal sample size $N/2$, where N is the total sample size. We first use sample I_1 to identify major and weak IVs, then use sample I_2 for causal inference. Suppose we have identified $p_1^{(1)}$ major IVs and $p_2^{(1)}$ weak IVs with the estimated effect size denoted as $\hat{\alpha}_1 = (\hat{\alpha}'_{1,M}, \hat{\alpha}'_{1,W})' \in \mathbb{R}^{p_1^{(1)}+p_2^{(1)}}$ when regressing exposure X_1 with the SNPs in G_1.

In sample I_2, MR-SPLIT combines the selected weak IVs into a new composite IV and uses it as an IV along with the major IVs:

$$\hat{G}_2 = (G_{2,M}, G_{2,W}\hat{\alpha}_{1,W}) \in \mathbb{R}^{\frac{N}{2}\times(p_1^{(1)}+1)} \tag{S2}$$

Then, we can apply the stage one of 2SLS in sample I_2 using these IVs and get the estimates of the exposure in sample I_2:

$$\hat{X}_2 = \hat{G}_2(\hat{G}_2'\hat{G}_2)^{-1}\hat{G}_2'X_2 = H_{\hat{G}_2}X_2,$$

where $H_X = X(X'X)^{-1}X'$ for any matrix X.

Similarly, we can also get the estimates of the exposure in sample I_1 by using sample I_2 to select the major and weak IVs:

$$\hat{X}_1 = \hat{G}_1(\hat{G}_1'\hat{G}_1)^{-1}\hat{G}_1'X_1 = H_{\hat{G}_1}X_1$$

Let $\hat{X} = \begin{pmatrix} \hat{X}_1 \\ \hat{X}_2 \end{pmatrix}$, $Y = \begin{pmatrix} Y_1 \\ Y_2 \end{pmatrix}$. In stage two, we get the estimate of MR-SPLIT as

$$\begin{aligned}\hat{\beta} &= (\hat{X}'\hat{X})^{-1}\hat{X}'Y \\ &= \beta + (X_1'H_{\hat{G}_1}X_1 + X_2'H_{\hat{G}_2}X_2)^{-1}(X_1'H_{\hat{G}_1}\varepsilon_{2,1} + X_2'H_{\hat{G}_2}\varepsilon_{2,2})\end{aligned}$$

and write $\varepsilon_2 = \begin{pmatrix} \varepsilon_{2,1} \\ \varepsilon_{2,2} \end{pmatrix}$.

For CFMR, it combines all selected IVs into a single IV. We use the subscript C to denote variables used in CFMR:

$$\hat{G}_{2,C} = (G_{2,M}\hat{\alpha}_{1,M}, G_{2,W}\hat{\alpha}_{1,W}) = G_2\hat{\alpha}_1 \in \mathbb{R}^{n\times 1} \tag{S3}$$

Similarly, in sample I_1, we combine G_1 and get:

$$\hat{G}_{1,C} = G_1\hat{\alpha}_2 \in \mathbb{R}^{n\times 1} \tag{S4}$$

For CFMR, let $\hat{G}_C = \begin{pmatrix} \hat{G}_{1,C} \\ \hat{G}_{2,C} \end{pmatrix}$, $X = \begin{pmatrix} X_1 \\ X_2 \end{pmatrix}$. Apply 2SLS on $\{X, Y, \hat{G}_C\}$ we get

$$\begin{aligned}\hat{\beta}_C &= (X'H_{\hat{G}_C}X)^{-1}X'H_{\hat{G}_C}Y \\ &= \beta + (X'H_{\hat{G}_C}X)^{-1}X'H_{\hat{G}_C}\varepsilon_2\end{aligned}$$

In the following, we will show that

$$var(\hat{\beta}) \leq var(\hat{\beta}_C)$$

where $\hat{\beta}$ denotes the estimate by MR-SPLIT. Since $var(\hat{\beta}) = (X_1'H_{\hat{G}_1}X_1 + X_2'H_{\hat{G}_2}X_2)^{-1}\sigma^2$, $var(\hat{\beta}_C) = (X'H_{\hat{G}_C}X)^{-1}\sigma^2$, to prove $var(\hat{\beta}) \leq var(\hat{\beta}_C)$, we need to show

$$\begin{aligned} & X_1'H_{\hat{G}_1}X_1 + X_2'H_{\hat{G}_2}X_2 \geq X'H_{\hat{G}_C}X \\ \iff\quad & X'\begin{pmatrix} H_{\hat{G}_1} & \\ & H_{\hat{G}_2} \end{pmatrix}X \geq X'H_{\hat{G}_C}X \\ \iff\quad & X'\left(\begin{pmatrix} H_{\hat{G}_1} & \\ & H_{\hat{G}_2} \end{pmatrix} - H_{\hat{G}_C}\right)X \geq 0 \end{aligned}$$

Hence, it is sufficient to show

$$\begin{pmatrix} H_{\hat{G}_1} & \\ & H_{\hat{G}_2} \end{pmatrix} - H_{\hat{G}_C} \succeq 0 \tag{S5}$$

where for any matrix X, $X \succeq 0$ means it is positive semi-definite.

Recall that $\hat{G}_C = \begin{pmatrix} \hat{G}_{1,C} \\ \hat{G}_{2,C} \end{pmatrix}$,

$$H_{\hat{G}_C} = \hat{G}_C(\hat{G}_C'\hat{G}_C)^{-1}\hat{G}_C' = \frac{1}{\hat{G}_{1,C}'\hat{G}_{1,C} + \hat{G}_{2,C}'\hat{G}_{2,C}}\begin{pmatrix} \hat{G}_{1,C}\hat{G}_{1,C}' & \hat{G}_{1,C}\hat{G}_{2,C}' \\ \hat{G}_{2,C}\hat{G}_{1,C}' & \hat{G}_{2,C}\hat{G}_{2,C}' \end{pmatrix}$$

Let $a = \hat{G}_{1,C}'\hat{G}_{1,C} + \hat{G}_{2,C}'\hat{G}_{2,C} \in \mathbb{R}$, it remains to show

$$\begin{pmatrix} H_{\hat{G}_1} - \frac{\hat{G}_{1,C}\hat{G}_{1,C}'}{a} & -\frac{\hat{G}_{1,C}\hat{G}_{2,C}'}{a} \\ -\frac{\hat{G}_{2,C}\hat{G}_{1,C}'}{a} & H_{\hat{G}_2} - \frac{\hat{G}_{2,C}\hat{G}_{2,C}'}{a} \end{pmatrix} \succeq 0 \tag{S6}$$

From Eq. S2 - S4, we can get

$$\begin{aligned}
H_{\hat{G}_2} =& \begin{pmatrix} G_{2,M} & G_{2,W}\hat{\alpha}_{1,W} \end{pmatrix} \left(\hat{G}_2'\hat{G}_2\right)^{-1} \begin{pmatrix} G_{2,M}' \\ \hat{\alpha}_{1,W}'G_{2,W}' \end{pmatrix} \\
=& \begin{pmatrix} G_{2,M} & G_{2,W}\hat{\alpha}_{1,W} \end{pmatrix} \begin{pmatrix} A_2 & B_2 \\ C_2 & D_2 \end{pmatrix} \begin{pmatrix} G_{2,M}' \\ \hat{\alpha}_{1,W}'G_{2,W}' \end{pmatrix} \\
=& G_{2,M}A_2G_{2,M}' + G_{2,W}\hat{\alpha}_{1,W}C_2G_{2,M}' + G_{2,M}B_2\hat{\alpha}_{1,W}'G_{2,W}' + G_{2,W}\hat{\alpha}_{1,W}D_2\hat{\alpha}_{1,W}'G_{2,W}' \\
\frac{\hat{G}_{2,C}\hat{G}_{2,C}'}{a} =& \frac{1}{a} \begin{pmatrix} G_{2,M} & G_{2,W} \end{pmatrix} \begin{pmatrix} \hat{\alpha}_{1,M}\hat{\alpha}_{1,M}' & \hat{\alpha}_{1,M}\hat{\alpha}_{1,W}' \\ \hat{\alpha}_{1,W}\hat{\alpha}_{1,M}' & \hat{\alpha}_{1,W}\hat{\alpha}_{1,W}' \end{pmatrix} \begin{pmatrix} G_{2,M}' \\ G_{2,W}' \end{pmatrix} \\
=& \frac{1}{a}(G_{2,M}\hat{\alpha}_{1,M}\hat{\alpha}_{1,M}'G_{2,M}' + G_{2,W}\hat{\alpha}_{1,W}\hat{\alpha}_{1,M}'G_{2,M}' \\
&+ G_{2,M}\hat{\alpha}_{1,M}\hat{\alpha}_{1,W}'G_{2,W}' + G_{2,W}\hat{\alpha}_{1,W}\hat{\alpha}_{1,W}'G_{2,W}')
\end{aligned} \tag{S7}$$

Therefore,

$$\begin{aligned}
H_{\hat{G}_2} - \frac{\hat{G}_{2,C}\hat{G}_{2,C}'}{a} =& G_{2,M}\left(A_2 - \frac{\hat{\alpha}_{1,M}\hat{\alpha}_{1,M}'}{a}\right)G_{2,M}' + G_{2,W}\hat{\alpha}_{1,W}\left(C_2 - \frac{\hat{\alpha}_{1,M}'}{a}\right)G_{2,M}' \\
&+ G_{2,M}\left(B_2 - \frac{\hat{\alpha}_{1,M}}{a}\right)\hat{\alpha}_{1,W}'G_{2,W}' + G_{2,W}\hat{\alpha}_{1,W}(D_2 - \frac{1}{a})\hat{\alpha}_{1,W}'G_{2,W}' \\
=& \begin{pmatrix} G_{2,M} & G_{2,W}\hat{\alpha}_{1,W} \end{pmatrix} \begin{pmatrix} A_2 - \frac{\hat{\alpha}_{1,M}\hat{\alpha}_{1,M}'}{a} & B_2 - \frac{\hat{\alpha}_{1,M}}{a} \\ C_2 - \frac{\hat{\alpha}_{1,M}'}{a} & D_2 - \frac{1}{a} \end{pmatrix} \begin{pmatrix} G_{2,M}' \\ \hat{\alpha}_{1,W}'G_{2,W}' \end{pmatrix} \\
=& \hat{G}_2Q_4\hat{G}_2'
\end{aligned} \tag{S8}$$

Similarly,

$$\begin{aligned}
H_{\hat{G}_1} - \frac{\hat{G}_{1,C}\hat{G}_{1,C}'}{a} =& \begin{pmatrix} G_{1,M} & G_{1,W}\hat{\alpha}_{2,W} \end{pmatrix} \begin{pmatrix} A_1 - \frac{\hat{\alpha}_{2,M}\hat{\alpha}_{2,M}'}{a} & B_1 - \frac{\hat{\alpha}_{2,M}}{a} \\ C_1 - \frac{\hat{\alpha}_{2,M}'}{a} & D_1 - \frac{1}{a} \end{pmatrix} \begin{pmatrix} G_{1,M}' \\ \hat{\alpha}_{2,W}'G_{1,W}' \end{pmatrix} \\
=& \hat{G}_1Q_1\hat{G}_1'
\end{aligned} \tag{S9}$$

Easily, we can also get

$$\begin{aligned}
-\frac{\hat{G}_{1,C}\hat{G}_{2,C}'}{a} =& -\frac{1}{a} \begin{pmatrix} G_{1,M} & G_{1,W}\hat{\alpha}_{2,W} \end{pmatrix} \begin{pmatrix} \hat{\alpha}_{2,M}\hat{\alpha}_{1,M} & \hat{\alpha}_{2,M} \\ \hat{\alpha}_{1,M}' & 1 \end{pmatrix} \begin{pmatrix} G_{2,M}' \\ \hat{\alpha}_{1,W}'G_{2,W}' \end{pmatrix} \\
=& -\frac{1}{a}\hat{G}_1Q_2\hat{G}_2'
\end{aligned} \tag{S10}$$

$$\begin{aligned}
-\frac{\hat{G}_{2,C}\hat{G}_{1,C}'}{a} =& -\frac{1}{a} \begin{pmatrix} G_{2,M} & G_{2,W}\hat{\alpha}_{1,W} \end{pmatrix} \begin{pmatrix} \hat{\alpha}_{1,M}\hat{\alpha}_{2,M} & \hat{\alpha}_{1,M} \\ \hat{\alpha}_{2,M}' & 1 \end{pmatrix} \begin{pmatrix} G_{1,M}' \\ \hat{\alpha}_{2,W}'G_{1,W}' \end{pmatrix} \\
=& -\frac{1}{a}\hat{G}_2Q_3\hat{G}_1'
\end{aligned} \tag{S11}$$

Apply Eq.S8 - S11 to Eq. S6, we have

$$\begin{pmatrix} \hat{G}_1 & \hat{G}_2 \end{pmatrix} \begin{pmatrix} Q_1 & Q_2 \\ Q_3 & Q_4 \end{pmatrix} \begin{pmatrix} \hat{G}_1' \\ \hat{G}_2' \end{pmatrix} \succeq 0 \tag{S12}$$

We now only need to show that

$$\begin{pmatrix} Q_1 & Q_2 \\ Q_3 & Q_4 \end{pmatrix} \succeq 0. \tag{S13}$$

We first show that

$$Q_4 = \begin{pmatrix} A_2 - \frac{\hat{\alpha}_{1,M}\hat{\alpha}_{1,M}'}{a} & B_2 - \frac{\hat{\alpha}_{1,M}}{a} \\ C_2 - \frac{\hat{\alpha}_{1,M}'}{a} & D_2 - \frac{1}{a} \end{pmatrix} \succ 0. \tag{S14}$$

Recall that

$$\begin{aligned}
\begin{pmatrix} A_2 & B_2 \\ C_2 & D_2 \end{pmatrix} =& (\hat{G}_2'\hat{G}_2)^{-1} \\
=& \begin{pmatrix} G_{2,M}'G_{2,M} & G_{2,M}'G_{2,W}\hat{\alpha}_{1,W} \\ \hat{\alpha}_{1,W}'G_{2,W}'G_{2,M} & \hat{\alpha}_{1,W}'G_{2,W}'G_{2,W}\hat{\alpha}_{1,W} \end{pmatrix}^{-1}
\end{aligned} \tag{S15}$$

Thus,

$$A_2 = (G'_{2,M}G_{2,M})^{-1} + \frac{(G'_{2,M}G_{2,M})^{-1}(G'_{2,M}G_{2,W}\hat{\alpha}_{1,W}\hat{\alpha}'_{1,W}G'_{2,W}G_{2,M})(G'_{2,M}G_{2,M})^{-1}}{\hat{\alpha}'_{1,W}G'_{2,W}(I-H_{G_{2,M}})G_{2,W}\hat{\alpha}_{1,W}},$$
$$B_2 = -\frac{(G'_{2,M}G_{2,M})^{-1}G'_{2,M}G_{2,W}\hat{\alpha}_{1,W}}{\hat{\alpha}'_{1,W}G'_{2,W}(I-H_{G_{2,M}})G_{2,W}\hat{\alpha}_{1,W}},$$
$$C_2 = -\frac{\hat{\alpha}'_{1,W}G'_{2,W}G_{2,W}(G'_{2,M}G_{2,M})^{-1}}{\hat{\alpha}'_{1,W}G'_{2,W}(I-H_{G_{2,M}})G_{2,W}\hat{\alpha}_{1,W}},$$
$$D_2 = \frac{1}{\hat{\alpha}'_{1,W}G'_{2,W}(I-H_{G_{2,M}})G_{2,W}\hat{\alpha}_{1,W}}.$$

Then,

$$\begin{aligned} D_2 - \frac{1}{a} =& \frac{1}{\hat{\alpha}'_{1,W}G'_{2,W}(I-H_{G_{2,M}})G_{2,W}\hat{\alpha}_{1,W}} - \frac{1}{\hat{G}'_{1,C}\hat{G}_{1,C}+\hat{G}'_{2,C}\hat{G}_{2,C}} \\ =& \frac{1}{\hat{\alpha}'_{1,W}G'_{2,W}(I-H_{G_{2,M}})G_{2,W}\hat{\alpha}_{1,W}} \\ & - \frac{1}{\hat{G}'_{1,C}\hat{G}_{1,C}+(G_{2,M}\hat{\alpha}_{1,M}+G_{2,W}\hat{\alpha}_{1,W})'(G_{2,M}\hat{\alpha}_{1,M}+G_{2,W}\hat{\alpha}_{1,W})} \end{aligned}$$

Since

$$\begin{aligned} &(G_{2,M}\hat{\alpha}_{1,M}+G_{2,W}\hat{\alpha}_{1,W})'(G_{2,M}\hat{\alpha}_{1,M}+G_{2,W}\hat{\alpha}_{1,W}) - \hat{\alpha}'_{1,W}G'_{2,W}(I-H_{G_{2,M}})G_{2,W}\hat{\alpha}_{1,W} \\ =&(G_{2,M}\hat{\alpha}_{1,M}+H_{G_{2,M}}G_{2,W}\hat{\alpha}_{1,W})'(G_{2,M}\hat{\alpha}_{1,M}+H_{G_{2,M}}G_{2,W}\hat{\alpha}_{1,W}) > 0, \end{aligned} \tag{S16}$$

we get $D_2 - \frac{1}{a} > 0$.
To prove S14, it is sufficient to show[1]

$$\begin{aligned} & A_2 - \frac{\hat{\alpha}_{1,M}\hat{\alpha}'_{1,M}}{a} - (B_2 - \frac{\hat{\alpha}_{1,M}}{a})(D_2-\frac{1}{Q})^{-1}(C_2 - \frac{\hat{\alpha}'_{1,M}}{a}) \succ 0 \\ \Longleftrightarrow & (A_2 - \frac{\hat{\alpha}_{1,M}\hat{\alpha}'_{1,M}}{a})(D_2 - \frac{1}{a}) - (B_2 - \frac{\hat{\alpha}_{1,M}}{a})(C_2 - \frac{\hat{\alpha}'_{1,M}}{a}) \succ 0 \\ \Longleftrightarrow & (a - \frac{1}{D_2})\tilde{A}_2^{-1} - \hat{\alpha}_{1,M}\hat{\alpha}'_{1,M} + \tilde{A}_2^{-1}\tilde{B}_2\tilde{C}_2\tilde{A}_2^{-1} - \hat{\alpha}_{1,M}\tilde{C}_2\tilde{A}_2^{-1} - \tilde{A}_2^{-1}\tilde{B}_2\hat{\alpha}'_{1,M} \succ 0 \end{aligned} \tag{S17}$$

where $\begin{pmatrix} \tilde{A}_2 & \tilde{B}_2 \\ \tilde{C}_2 & \tilde{D}_2 \end{pmatrix} = \hat{G}'_2\hat{G}_2$. The left side of Eq. S17 can be obtained as $(a - \frac{1}{D_2})\tilde{A}_2^{-1} + (\tilde{A}_2^{-1}B_2 - \hat{\alpha}_{1,M})(\tilde{A}_2^{-1}B_2 - \hat{\alpha}_{1,M})'$, which is easy to verify to be a positive definite matrix.
To prove Eq. S13, now we only need to prove

$$Q_1 - Q_2Q_4^{-1}Q3 \succeq 0. \tag{S18}$$

From Eq. S14, we have

$$\begin{aligned} Q_4^{-1} &= \left((\hat{G}'_2\hat{G}_2)^{-1} - \frac{1}{a}\begin{pmatrix} \hat{\alpha}_{1,M} \\ 1 \end{pmatrix}\begin{pmatrix} \hat{\alpha}'_{1,M} & 1 \end{pmatrix}\right)^{-1} \\ &= \left((\hat{G}'_2\hat{G}_2)^{-1} - \frac{1}{a}b_2b'_2\right)^{-1} \\ &= \hat{G}'_2\hat{G}_2 - \frac{\hat{G}'_2\hat{G}_2b_2b'_2\hat{G}'_2\hat{G}_2}{b'_2\hat{G}'_2\hat{G}_2b_2 - a}, \end{aligned} \tag{S19}$$

where $b_2 = \left(\hat{\alpha}'_{1,M}, 1\right)'$, and the third equation utilizes the Woodbury matrix identity. Similarly, let $b_1 = \left(\hat{\alpha}'_{2,M}, 1\right)'$,

$$Q_1 = (\hat{G}'_1\hat{G}_1)^{-1} - \frac{1}{a}b_1b'_1, \tag{S20}$$

$$Q_2 = -\frac{1}{a}b_1b'_2, \tag{S21}$$

$$Q_3 = -\frac{1}{a}b_2b'_1. \tag{S22}$$

Substituting Eq. S19 -S22 into Eq. S18, we get

$$\begin{aligned}
&(\hat{G}'_1\hat{G}_1)^{-1} - \frac{1}{a}b_1b'_1 - \frac{1}{a^2}b_1b'_2(\hat{G}'_2\hat{G}_2 - \frac{\hat{G}'_2\hat{G}_2b_2b'_2\hat{G}'_2\hat{G}_2}{b'_2\hat{G}'_2\hat{G}_2b_2 - a})b_2b'_1 \succeq 0 \\
\Longleftrightarrow &(\hat{G}'_1\hat{G}_1)^{-1} - b_1(\frac{1}{a} + \frac{1}{a^2}b'_2(\hat{G}'_2\hat{G}_2 - \frac{\hat{G}'_2\hat{G}_2b_2b'_2\hat{G}'_2\hat{G}_2}{b'_2\hat{G}'_2\hat{G}_2b_2 - a})b_2)b'_1 \succeq 0 \\
\Longleftrightarrow &(\hat{G}'_1\hat{G}_1)^{-1} - b_1(\frac{1}{a} + \frac{\hat{G}'_{2,C}\hat{G}_{2,C}}{a^2} - \frac{(\hat{G}'_{2,C}\hat{G}_{2,C})^2}{a^2\hat{G}'_{2,C}\hat{G}_{2,C} - a^3})b'_1 \succeq 0 \\
\Longleftrightarrow &(\hat{G}'_1\hat{G}_1)^{-1} - \frac{1}{\hat{G}'_{1,C}\hat{G}_{1,C}}b_1b'_1 \succeq 0
\end{aligned} \tag{S23}$$

Eq. S23 has a very similar structure as Eq. S14. It can be written as

$$\begin{pmatrix} A_1 - \frac{\hat{\alpha}_{2,M}\hat{\alpha}'_{2,M}}{\hat{G}'_{1,C}\hat{G}_{1,C}} & B_1 - \frac{\hat{\alpha}_{2,M}}{\hat{G}'_{1,C}\hat{G}_{1,C}} \\ C_1 - \frac{\hat{\alpha}'_{2,M}}{\hat{G}'_{1,C}\hat{G}_{1,C}} & D_1 - \frac{1}{\hat{G}'_{1,C}\hat{G}_{1,C}} \end{pmatrix} \succeq 0, \tag{S24}$$

which can be easily verified. This completes the proof of the theorem. □

References

[1] Zhang F. The Schur complement and its applications. vol. 4. Springer Science & Business Media; 2006.
