# Supplementary material for: MR-SPLIT: A novel method to address selection and weak instrument bias in one-sample Mendelian randomization studies
Source: PLoS Genet. 2024 Sep 6;20(9):e1011391. doi: 10.1371/journal.pgen.1011391 (PMC11410202; doi:10.1371/journal.pgen.1011391)
Supplement: Supplementary file 2 [file pgen.1011391.s002.pdf]

We greatly appreciate the three reviewers for their insightful comments and suggestions that greatly helped improve the manuscript. Below we list our point-by-point responses to the reviewers' comments. All the major changes in the manuscript are highlighted with red fonts. Our responses to the reviewers' comments are listed below.

Response to Reviewer #1

The manuscript presented, MR-SPLIT, a novel method to address selection and weak instrument bias in one-sample MR analysis. The authors first provided the theoretical justification in terms of the efficiency for the causal effect estimate, and then conducted various simulations, parallelized with real data applications, to illustrate the advantage of MR-SPILIT over other competing methods. Overall, the manuscript is of great interest and well-written, I just have the following comments for further improvement.

R: Thank you for your positive comments about our work.

1: Given that including more correlated IVs, rather than only independent IVs through LD clumping or pruning, can increase the power of MR analysis. I wonder whether MR-SPLIT can be developed for correlated IVs. I suggested the authors to discuss this.

R: Thank you for the comment! If we understand your comment correctly, we believe you are referring to the inclusion of IVs with both uncorrelated and correlated horizontal pleiotropic effects. Indeed, this is a valuable point. Methods have been proposed in recent years to incorporate these types of IVs, which may potentially increase power and decrease bias. Including them in our work is nontrivial and will be considered in our future work. We have discussed this in the discussion section and cited relevant literature (see page 19).

2. It seems that there is no gold standard to determine either the major or the weak IVs. For fixed number of IVs, dose the performance of MR-SPLIT rely on the ratio of the number of major IVs to the number of weak IVs.

R: Thank you for the comment! Yes, there is no gold standard to determine major and weaker IVs. The extreme case is to combine all IVs, regardless of major or weaker ones, to form a composite one, as what the CFMR does. Such a composite IV is also called an allelic score and is similar to the polygenic risk score (PRS) in risk prediction. When using a smaller F threshold, it leads to more major IVs which may lead to a larger bias (but a smaller variance). On the other hand, using a larger F threshold will yield fewer major IVs, which may lead to a smaller bias (but a larger variance). So essentially deciding the threshold is a problem of dealing with bias-variance tradeoff.

In our simulation study, we found that F>30 is a reasonable threshold which yields reasonable separation of major and weaker IVs (also depends on SNP heritability). As revealed by the simulation studies, the declaration of major IVs may vary under different F thresholds and under different sample sizes and SNP heritability levels. In real applications, the F > 30 threshold can be relaxed under a small sample size and low heritability level. We have discussed this in the discussion section (see page 19). We also mentioned that other criteria other than the F value could

be used. Any statistical measure capable of ranking the effect sizes of the selected IVs could be considered for enhancing the robustness and effectiveness of our approach.

3: In the real data analysis, it seems that the power advantage of MR-SPLIT over CFMR is not that obvious, and CFMR can even produce much smaller p value than MR-SPLIT. I suggested the authors to investigate the possible reasons. For example, are there any IV associated with the outcome? If so, what happened if we remove them from the analysis. Indeed, this can partly alleviate the issue of pleiotropy.

R: Thank you for the comment! This is a very good observation. The result could be due to various reasons such as a small sample size or the presence of the potential pleiotropic effect. Identifying and removing IVs with pleiotropic effect is a challenging task. Methods dealing with pleiotropic IVs have been extensively studied in two-sample MR analysis with GWAS summary statistics. However, they are largely lacking in one-sample MR analysis. We will develop methods to identify and remove IVs with potential pleiotropic effecst in our future work.

4. In the real data analysis, what is the criteria to determine the major IVs or the weak IVs? Again, what happened if we changed the ratio of the number of major IVs to the number of weak IVs.

R: In the real data analysis, we use the same criteria for choosing major IVs as we did in the simulation (partial F>30). The ratio of the number of major IVs is not manually set but is automatically selected by the method itself based on the strength of the IVs. To provide more clarity, we have added additional detail in the second paragraph of section 4.2 (see page 14). We also tried to lower the partial F threshold to 20 which yields more major IVs than the F>30 threshold. We did see the p-value for MR-SPLIT improves a little bit (from 5.9E-05 to 2.9E-06). However, the improvement is not very significant.

*5. There are some typos in the manuscript, For example, in line 4 of the 4.3 section, p=4.6*10(-12). I suggested the authors to double check the whole manuscript again.*

R: Thank you very much for pointing out this error. It has been corrected, and the entire manuscript has been proof-read.

Response to Reviewer #2

This manuscript proposed a novel method named as Mendelian Randomization with adaptive Sample-sPLitting with cross-fitting InstrumenTs (MR-SPLIT) to address IV selection issue and weak instrument bias under the 2SLS IV regression framework.
Overall, the paper is well-written and the methods are nice outlined both in the main text and in supplementary materials. Below are my comments and questions:

R: We greatly appreciate your positive comments about our work.

Introduction:
P2. The author explained the assumptions of IVs in the MR analysis. The violation of the relevance assumption leads to a 'weak instrument' issue. Please describe why the challenge of IV selection should also be considered when this problem was first mentioned.

R: Thanks for the comment. Weak IVs can lead to bias, and this bias could be further amplified when the same sample is used twice for both IV selection and causal effect estimation (double dipping), though this is a common practice in one-sample MR analysis. In Section 2 of the supplementary file, the simulation results show that using the same data (the whole sample) for both IV selection and causal effect estimation, both LIML and 2SLS could amplify bias compared to using half for selection and the other half for estimation (indicated by LIML_w and 2SLS_w). We have added a few sentences to further address this issue (see page 2).

P5 and P7. The author only introduced methods that handled the weak instrument issue. Are there any methods that consider the IV selection issue? Please add some citations.

R: Upon our careful review, we found that currently there are no methods specifically addressing this issue. It is only mentioned as a potential problem in some papers. We have cited some papers (in the Introduction section) and they are:

- Burgess S, Smith G D, Davies N M, et al. Guidelines for performing Mendelian randomization investigations: update for summer 2023. Wellcome open research, 2019, 4.
- Jiang T, Gill D, Butterworth A S, et al. An empirical investigation into the impact of winner's curse on estimates from Mendelian randomization. International Journal of Epidemiology, 2023, 52(4): 1209-1219.

P8. Please summarize how this method addressed the bias of IV selection.

R: The key to obtaining good estimation results with the 2SLS method is to use the selected IVs to achieve a fit as good as possible for the exposure. The bias in IV selection could arise from two aspects:

1. The usage of a p-value threshold criterion in the selection of IVs is somewhat arbitrary and lacks robust justification.
2. Using the same sample for both IV selection and estimation could lead to biased causal effect estimates.

In our MR-SPLIT method, we randomly split the sample into two non-overlapping subsamples: one for IV selection and the other for obtaining the cross-fitting exposure values based on the selected IVs. This is a key step to address the IV selection bias issue, in addition to separating major and weak IVs and obtaining cross-fitting exposures for further causal effect estimation.

Methods:

In section 2.1.1 P3. Please describe the definition of partial F-statistics and explain why to use partial F-statistics as criteria.

R: Thank you for pointing this out. We have updated the definition in Section 2.1.1 (see page 5).

In section 2.1.2. Please explain how to use I_1 and I_2 to select major and weak IVs, respectively. Could you please explain whether the weak IVs used to construct composite IV are the same as the IVs obtained in sample I_1?

R: The IVs selected from *I_1* and *I_2* are different in different subsamples. We use weak IVs selected from *I_1* to construct composite IV in *I_2*. We have also added an additional explanation in section 2.1.2 to make it clear (see page 5)

In Remark 2. The author described that MR-SPLIT benefits from a 2-fold sample splitting. It selects and separates major and weak IVs in I_1, then forms the cross-fitting composite IV in I_2. Please explain why 2-fold sample splitting can deal with the weak instrument bias and IV selection issue.

R: Thanks for the comment. To decrease the bias caused by the weak instrument bias, we combine the many weak IVs into only one composite IV to summarize the information and decrease the number of IVs to do the estimation. To address the IV selection issue, we do the IV selection and effect estimation in different samples. We opted for a 2-fold approach over others because our method relies both on the accuracy of IV selection and the precision of obtaining the cross-fitted exposure. When the number of folds is larger than 2, there always exists an unbalanced situation where a larger (smaller) sample is used for IV selection and a smaller (larger) sample is used to get the cross-fitted exposure. In either case, the use of the smaller sample will inevitably run into the small sample issue. We experimented with 5-fold and 10-fold splitting approaches and found that neither outperformed the 2-fold method. Thus, we chose the 2-fold splitting method.

In section 2.1.5 algorithm. How to define the value of L random split in the simulations studies and real data analysis?

R: Thanks for the comment. We discussed the choice of *L* in Section 3.4 which is based upon analyzing the stabilization trend in the type I error and power. For example, we found that when N=500, L=50 achieves stable results in the sense that the type I error and power get stable. In principle, more splits should lead to more stable results. However, more splits will consume more computational power and time. To balance the computational time and accuracy, the number of splits is chosen as the one that stabilizes the type I error and power. We chose L=50 as a conservative number of splits. As revealed by simulation results, a smaller number of splits can be chosen when the sample size is large and the SNP heritability level is high, especially when computational time is a concern.

In the real data analysis, we chose L=50.

Simulation Study:
In section 3.1. The author can determine what criteria to distinguish the major and weak IVs by conducting simulations. Please describe the value of the threshold used in the real data analysis and how to determine this threshold.

R: Thanks for the comment. In all simulations and real data analyses, we used a threshold of partial F>30 to distinguish major IVs. We detailed the reasons for choosing this threshold and offered recommendations in Section 3.1. Again, the use of F>30 is not a golden standard for declaring major IVs. It also varies considering the sample size and SNP heritability level in real applications. For example, when the sample size is small (e.g., *N*=500 or less), one might want to reduce this threshold and use F>20 or even F>10 as the threshold to choose major IVs. One can also get an estimate of the genomewide SNP heritability using software such as GCTA and choose an

appropriate F threshold based on the estimated heritability. We have added a few sentences to discuss this in the discussion section (see page 18).

In sections 3.1 and 3.2. Please add more details to explain how to randomly generate 300 SNPs. Do you generate the simulated datasets following the model (1) in section 2? Are these SNPs independent with each other? Do you consider the LD matrix when generating the SNPs?

R: Thank you for pointing this out. We have added more detailed information about generating the SNPs and the data (see page 8). We did not consider LD between SNPs. In real data analysis, we had the LD pruning step to remove SNPs with LD. This is a common practice to select SNP IVs in MR analysis.

P2. Please add more details on conducting simulations to check the impact of selection bias, including specific methods for sample division.

R: We conducted two versions of the 2SLS and LIML analyses. In version 1, we used the whole sample for both IV selection and estimation. In contrast, version 2 involved randomly dividing the whole sample into two parts, using one half for IV selection and the other half for estimation. The comparison results are shown in the supplementary file and are also discussed in Section 3.2. The results in Section 2 in the supplemental file indicated by "_w" refer to the results using the whole sample for both IV selection and causal effect estimation. We also included a few sentences to clarify this (see page 10). LIML is developed to address the weak IV issue. However, it still leads to biased estimate if using the same data for both IV selection and effect estimation (see LIML_w). This demonstrates the problem of IV selection bias and highlights the importance of sample splitting to address the IV selection bias issue.

Case Study:
P2. Please explain the reason for using a LASSO-projection method. It would be helpful to provide some numerical results to show that this one is better than LASSO algorithm to select and estimate SNP effects.

R: We have discussed this in the discussion section about the benefits of the LASSO-projection method. We did observe in the simulations that the LASSO method selected more IVs compared to the LASSO-projection method. Many of these IVs selected by LASSO demonstrate extremely weak or no association with the exposure (reflected by partial p-values). This makes sense since the LASSO method tends to generate a prediction model that minimizes the prediction variance (with the price of increasing the model bias). A model with better prediction performance (with a larger bias) may not be the one that has all variables showing statistical significance. On the other hand, the LASSO-projection method is to debias the LASSO estimates, hence reduces bias and gives uncertainty quantification of individual SNP variables. As a result, the number of IVs selected by the LASSO projection method will be smaller than the regular LASSO method. In our real data analysis, the regular LASSO method gave a larger number of IVs than the LASSO-projection method did (double the IVs selected by the LASSO-projection method in many cases). Many of these IVs may violate the relevance assumption and may lead to a larger p-value when testing the causal effect. This issue is further amplified when the sample size is small.

In our real data analysis, using IVs selected by the regular LASSO algorithm gave a p-value larger than the one with the LASSO-projection method. Thus, we adopted the LASSO-projection method in real data analysis. It's worth noting that the LASSO-projection method takes more time than the LASSO method. When the computational power is limited, one can use the ridge-projection method in high-dimensional inference which is much faster than the LASSO-projectin method, though its accuracy is not as high as the LASSO-projection method.

We have also conducted additional simulations to compare these two methods, and we have included the results in the supplementary file (see section 6).

Please describe the value of the partial F statistic threshold and the reason.

R: We choose partial F>30 as the threshold to select the major IVs. This decision comes from our simulation results in section 3.1. We have added this to make it clear (see page 15). We also discussed the impact of different thresholds in the discussion section (see page 18).

Could you provide any references that confirm the causal relationship between eGFR and aTRH? If available, please include the citations.

R: To date, the causal relationship between CKD (assessed using eGFR or uACR) and aTRH has yet to be established, with no reference available confirming the causal relationship. However, our method detected a causal link between these measures and aTRH, consistent with the CFMR results. This leads us to conclude a causal connection between CKD and aTRH, though further biological verification is needed.

In real data analysis, the author only compares the MR-SPLIT with CFMR. Please add more results to compare with LIML and 2SLS.

R: Thanks for the comment. We did compare our method with 2SLS (in addition to CFMR) in the real data application. Compared to MR-SPLIT and CFMR, the estimators from 2SLS are quite biased. LIML is only applicable to a continuous response. Thus, we did not compare with LIML as aTRH is a binary variable.

Response to Reviewer #3

The author introduces MR-Split, an interesting extension of the work of Denault and colleagues on CFMR. The limitations of the CFMR are well presented, and the approach proposed by the author is sound and seems to address correctly the limitations of CFMR. They even provide theoretical arguments for MR split superiority compared to CFMR.

I am positive about accepting this paper, but I have a minor revision.

R: Thank you for your careful review and positive comment about our work.

Comments:

1) The paper needs some polishing/sharpening. For example, at the start of section two, the authors say, "assume the following 2SLS" model. 2SLS is an estimation method for this kind of model but not a model by itself. This model is an SEM. Another example in the supplementary

material on page 14 is that the authors wrote, "To prove S14, we need to show ???" This is obviously a latex mistake regarding the references. It would be good if this kind of typos/impreciseness were corrected for the next version.

R: Thank you for the comments! We have proofread the entire manuscript and corrected the errors.

2) Regarding Figure 5, I appreciate that the authors point out the lack of control of type one error when increasing the number of splits in a small sample size. I suspect that some simple bootstrapping method could be used to correct this bias. If this is not possible, I still agree that the power gain is worth a slight increase in type I error. Furthermore, it would be good to have a smoother estimate of the type I error. Could the authors perform more simulations(given that this is for a small sample, I suspect that should be doable.

R: Thank you so much for the suggestion. Currently, our simulations in Section 3.4 are repeated 1,000 times. We tried to increase the simulation runs to 5,000 and did see a smoother estimate of the type I error. We have updated the figures and added descriptions in the manuscript.

Again, we greatly appreciate all the comments and suggestions raised by the reviewers. Thank you!
