# Supplementary material for: MR-SPLIT: A novel method to address selection and weak instrument bias in one-sample Mendelian randomization studies
Source: PLoS Genet. 2024 Sep 6;20(9):e1011391. doi: 10.1371/journal.pgen.1011391 (PMC11410202; doi:10.1371/journal.pgen.1011391)
Supplement: Supplementary file 3 [file pgen.1011391.s003.pdf]

We greatly appreciate the two reviewers for their insightful comments and suggestions that greatly helped to improve the manuscript. Below we list our point-by-point responses to the reviewers' comments. All the major changes in the manuscript are highlighted with red fonts. Our responses to the reviewers' comments are listed below.

Response to Reviewer #1:

The authors have addressed all my comments.

R: Thank you for your positive feedback. We are pleased to hear that we have addressed all your comments.

Response to Reviewer #2:

Summary:

This manuscript proposed a novel method named as Mendelian Randomization with adaptive Sample-sPLitting with cross-fitting InstrumenTs (MR-SPLIT) to address IV selection issue and weak instrument bias under the 2SLS IV regression framework.

Overall, the paper is well revised, both in the main text and in supplementary materials based on the major comments, but I have the following minor suggestions:

R: Thank you for your positive feedback and for acknowledging the revisions based on the major comments. We appreciate your minor suggestions and have addressed them accordingly as detailed below.

Methods

1. Please include brief descriptions in Figure 1 to outline the workflow of MR-SPLIT and refine this flow chart. Specifically, please adjust the size of the rectangles (the third row).

R: Thank you for your valuable suggestion. We have added a brief description to describe the MR-SPLIT procedure and adjusted the size of the rectangles in the 3rd row to make the two rectangles the same size.

2. In Sections 2.1.2 and 2.1.3, please describe the notations α ̂and {G_M,G ̂_W} clearly.

R: Thank you for your valuable suggestion. We have revised Sections 2.1.2 and 2.1.3 to clearly describe the notations $\hat{\alpha}$ and $\{G_M, \hat{G}_W\}$.

3. In Section 2.1.3 Remark 1, please explain the notation n_k in G ̃_(k,n_k×1).

R: Thank you for your valuable suggestion. We have revised Section 2.1.3 Remark 1 to clearly describe the notation n_k.

Simulations

1. In Section 3.1.1, please add more details and explain the processes for generating independent SNPs, the exposure, and the outcome clearly (i.e., the value of β and error terms).

R: Thank you for your valuable suggestion. We did not simulate the outcome in Section 3.1.1 because this part primarily concerns the first stage of the 2SLS method, which is unrelated to the outcome. We have explained how the SNPs were simulated and described the distribution of error terms.

2. Please add the explanations of the last column (Noises) in the caption of Table 1 and Section 3.1.1.

R: Thank you for your valuable suggestion. We have added explanations for the last column (Noises) in Table 1 and in Section 3.1.1.

3. In Section 3.1.2, the first line has an extra right parenthesis on page 11.

R: Thank you for pointing this out. We have corrected the extra right parenthesis on the first line of Section 3.1.2 on page 11.

Again, we greatly appreciate the insightful comments and suggestions from the reviewer. Thank you!
